# Aurora B prevents aneuploidy via MAD2 during the first mitotic cleavage in oxidatively damaged embryos

**DOI:** 10.1111/cpr.12657

**Published:** 2019-07-01

**Authors:** Jiena Li, Siyao Ha, Zhiling Li, Yue Huang, En Lin, Wanfen Xiao

**Affiliations:** ^1^ Reproductive Center, The First Affiliated Hospital of Shantou University Medical College Shantou University Shantou China; ^2^ Laboratory for Reproductive Immunology, Hospital & Institute of Obstetrics & Gynecology Fudan University Shanghai Medical College Shanghai China

**Keywords:** Aurora B, chromosome aneuploid, in vitro fertilization, oxidative stress, spindle

## Abstract

**Objectives:**

A high rate of chromosome aneuploidy is exhibited in in vitro fertilization (IVF)‐derived embryos. Our previous experiments suggested that reactive oxygen species (ROS) can activate Mad2, a key protein in the spindle assembly checkpoint (SAC), and delay the first mitotic, providing time to prevent the formation of embryonic aneuploidy. We aimed to determine whether mitotic kinase Aurora B was involved in the SAC function to prevent aneuploidy in IVF‐derived embryos.

**Materials and Methods:**

We analysed aneuploidy formation and repair during embryo pre‐implantation via 4ʹ,6‐diamidino‐2‐phenylindole (DAPI) staining and karyotype analysis. We assessed Aurora B activation by immunofluorescence and investigated the effect of Aurora B inhibition on embryo injury‐related variables, such as embryonic development, ROS levels, mitochondrial membrane potential and γH2AX‐positive expression.

**Results:**

We observed the expression and phosphorylation of Thr232 in Aurora B in oxidative stress‐induced zygotes. Moreover, inhibition of Aurora B caused chromosome mis‐segregation, abnormal spindle structures, abnormal chromosome number and reduced expression of Mad2 in IVF embryos. Our results suggest that Aurora B causes mitotic arrest and participates in SAC via Mad2 and H3S10P, which is required for self‐correction of aneuploidies.

**Conclusions:**

We demonstrate here that oxidative stress–induced DNA damage triggers Aurora B‐mediated activation of SAC, which prevents aneuploidy at the first mitotic cleavage in early mouse IVF embryos.

## 1 INTRODUCTION

The spindle assembly checkpoint (SAC) represents a surveillance mechanism that ensures the accurate separation of sister chromatids during early mitosis.^1^, ^2^ Aurora kinase B (Aurora B) is a member of the chromosomal passenger complex and is required for proper regulation of chromosome alignment, cytokinesis and kinetochore‐microtubule interactions. It is believed to be involved in the maintenance of SAC signalling.^3^, ^4^, ^5^ Thus, Aurora B is important for proper chromosome separation and the maintenance of genomic integrity, remaining active until all kinetochores are correctly attached to spindle microtubules during mitosis.^6^

Early embryos in in vitro fertilization (IVF) exhibit a high rate of chromosome aneuploidy,^7^, ^8^ which may explain the high developmental failure rates during the culture of embryos in the IVF setting.^9^, ^10^ In assisted reproductive technology, in vitro culture does not completely simulate the in vivo environment. For example, in vitro cultures may be influenced by culture fluid (pH and contained substances), light, temperature and gas phase, leading to a significant increase in reactive oxygen species (ROS) concentrations in embryonic cells.^11^, ^12^ Thus, oxidative stress–induced DNA damage is inevitable during in vitro culture, thereby disrupting the development of embryonic cells.^13^, ^14^ The main reason for the arrest of embryonic development is aneuploidy,^15^ in which the developing offspring exhibits chromosome mis‐segregation.^16^, ^17^ After fertilization, mitotic errors are associated with the formation of aneuploidy in pre‐implantation embryos.^18^, ^19^

In previous work, we found that 0.03 mmol/L hydrogen peroxide(H_2_O_2_) administered at 7 hours post‐insemination (hpi) (G_1_ phase) was the minimum concentration necessary to establish a model system that simulates the clinically inevitable oxidative stress experienced by IVF embryos^20^ and resulted in an increase in sex chromosome aneuploidy in mouse IVF embryos.^21^ We previously used isobaric tags for relative and absolute quantitation (iTRAQ) experiments to reveal that oxidative stress inhibited the expression of Raf kinase inhibitory protein (RKIP), which caused a defect in the mitotic SAC by reducing the centromere localization of Aurora B in some H_2_O_2_‐treated zygotes; this may be one of the important reasons for chromosomal aneuploidies in IVF‐derived embryos.^22^, ^23^ However, the role of Aurora B in zygotes subjected to oxidative stress‐induced injury remains unclear. In this study, we aimed to determine whether mitotic kinase Aurora B was involved in SAC function to prevent aneuploidy in IVF‐derived embryos. Furthermore, we evaluated the mechanism underlying the role of Aurora B during the first mitotic cleavage period.

## 2 MATERIALS AND METHODS

### 2.1 Experimental animals

Adult Kun‐Ming mice (3‐6 weeks old) were purchased from Beijing Vital River Laboratory Animal Technology and treated in compliance with the Guide for the Care of Use of Laboratory Animal by the US National Institutes of Health (NIH Publication number 85‐23, revised 1996) and the rules of the National Animal Protection of China. All experimental protocols were approved by the Laboratory Animal Ethics Committee of our institution (SUMC2014‐014). This study was approved by the Institutional Animal Care and Use Committee of Shantou University Medical College.

### 2.2 Reagents and media

Rabbit anti‐Aurora B antibodies (ab2254), rabbit anti‐MAD2L2 [EPR13657] antibodies (ab180579), rabbit anti‐phospho‐histone H3 (Ser10) antibodies and rabbit anti‐phospho‐histone H2AX (phospho‐S139) antibodies (ab2893) were purchased from Abcam (Cambridge, UK). AZD1152‐HQPA was purchased from Abcam. Mouse anti‐Aurora B antibodies (3F11) were obtained from R&D (Chicago, IL, USA). Anti‐Aurora B (phospho‐Thr232) antibodies were obtained from Cell Signaling Technology (Danvers, MA, USA). Goat anti‐rabbit Alexa Fluor 488 and goat anti‐mouse IgG H&L (Alexa Fluor 647) were obtained from Abcam. Human tubal fluid (HTF) was obtained from Sage Science (Beverly, MA, USA). 4ʹ,6‐diamidino‐2‐phenylindole (DAPI) (10 μg/mL) was obtained from Solarbio (China).

### 2.3 Collection of spermatozoon and oocytes, IVF and culture of zygotes

As described in our previous studies,^20^ spermatozoon was collected from the murine cauda epididymis and incubated in capacitation medium (HTF medium containing 1.5% BSA) at 37°C in a 5% CO_2 _incubator for 1 hour. Female mice were induced to superovulate by consecutive intraperitoneal injections of 10 IU pregnant mare serum gonadotropin and 10 IU human chorionic gonadotropin (HCG) 48 hours apart, followed by euthanization at 13‐15 hours after HCG administration to obtain cumulus oocytes from the oviducts. Cumulus oocytes were collected in PBS prewarmed to 37°C, followed by transfer to prepared 37°C fertilization liquid (HTF medium containing 0.4% BSA) under oil containing 10 μL capacitated spermatozoon. Samples were then incubated at 37°C for 6 hours in a 5% CO_2_ incubator to permit fertilization. Zygotes were washed three times and cultured in a new medium.

### 2.4 Mouse Zygote Model for Oxidative DNA Damage

According to our previous studies,^24^ in preliminary experiments, zygotes (7 hpi) were placed in culture medium with concentrations of 0.03 mmol/L H_2_O_2_ for 30 minutes and incubated at 37°C in an atmosphere containing 5% CO_2_ to obtain a model of oxidative DNA damage in mouse zygotes.

### 2.5 Immunofluorescence staining for Aurora B, phospho‐Aurora B(Thr232), H3S10P, MAD2L1, α‐tubulin and γH2AX

Immunofluorescence was performed as described previously.^20^ Zygotes were collected at different time points to detect the activation of various proteins. γH2AX was detected at 17.0‐18.0 hpi, Aurora B and phospho‐Thr232 Aurora B were detected at 20.5‐21.5 hours, MAD2L1 and Aurora B were detected at 21.5‐22.5 hpi, and H3S10P and Aurora B were detected at 18‐24 hpi in the control and treated groups. FITC‐conjugated anti‐α‐tubulin antibodies (mouse monoclonal antibodies) were provided by Sigma (Germany).

### 2.6 Aurora B inhibition studies

The Aurora B inhibitor, AZD1152‐HQPA, was dissolved in dimethyl sulfoxide (DMSO; final concentration of DMSO in the experiments did not exceed 0.1%). The inhibition rate of Aurora B was calculated as the difference between the Aurora B positivity rate in the H_2_O_2_‐treated group and the AZD1152‐HQPA/H_2_O_2_‐treated group, divided by the Aurora B positivity rate in the H_2_O_2_‐treated group.

### 2.7 Determination of ROS and MMP

Intracellular ROS levels and MMP were detected by DCFH‐DA and JC‐1. A stock solution of DCFH‐DA (1 × 10^−3^ mol/L in DMSO) was added to the embryo culture medium to a final concentration of 10 μmol/L, and JC‐1 was diluted in PBS to a final concentration of 1.25 μmol/L. As described in an earlier report,^20^ Image Pro Plus 6.0 (Media Cybernetics Inc, Bethesda, MD, USA) was used to quantify the fluorescence.

### 2.8 DAPI staining

The zona pellucida was removed from zygotes with Tyrode's solution. After washing with PBS containing 0.05% Tween 20 (PBST) three times for 5 minutes each, the zygotes were fixed in 4% paraformaldehyde for 30 minutes, mounted on polylysine‐coated slides and washed three times again with PBST. Zygotes were then permeabilized in PBST supplemented with 0.5% Triton X‐100 for 30 minutes at room temperature, counterstained with DAPI at room temperature for 30 minutes, washed three times and mounted with antifade fluorescence‐mounting medium before a coverslip was added.

### 2.9 Karyotype analysis

Zygotes were placed in a hypotonic solution (0.068 mol/L KCI) and treated at 37°C for 25 minutes. At each time point, 10‐15 hypotonically treated fertilized eggs were transferred to fixative I (methanol: glacial acetic acid: distilled water = 5:1:2.5) for 2‐3 minutes. When the volume increased and the colour changed from the tan colour present at the time of initial fixation, the slides were removed. After spreading the fixative I extract with the eggs on the slide, the cells were gently covered with fixative II (methanol: glacial acetic acid = 3:1), fixed again, firmly attached to the slide and then further fixed in fixative solution for 5 minutes. Subsequently, it was treated with fixative III (methanol: glacial acetic acid: distilled water = 3:3:1) for 1 minute, the preparation was carefully and slowly removed from fixative III, air‐dried, stained with 10% Giemsa working solution (0.067 mol/L phosphate buffer) for 30 minutes and examined by microscopy.

### 2.10 Statistical analysis

Results were collected from at least three independent experiments, and data were analysed using SPSS 17.0 software (SPSS Inc, Chicago, IL, USA). Data shown as percentages were analysed using chi‐square tests. Values expressed as means ± standard deviations were compared using Student's *t* tests. Differences with *P* < 0.05 were considered statistically significant.

## 3 RESULTS

### 3.1 Expression and subcellular localization of Aurora B during different phases in the cell cycle and oxidative stress–induced DNA damage in mouse embryos

**Figure 1:**
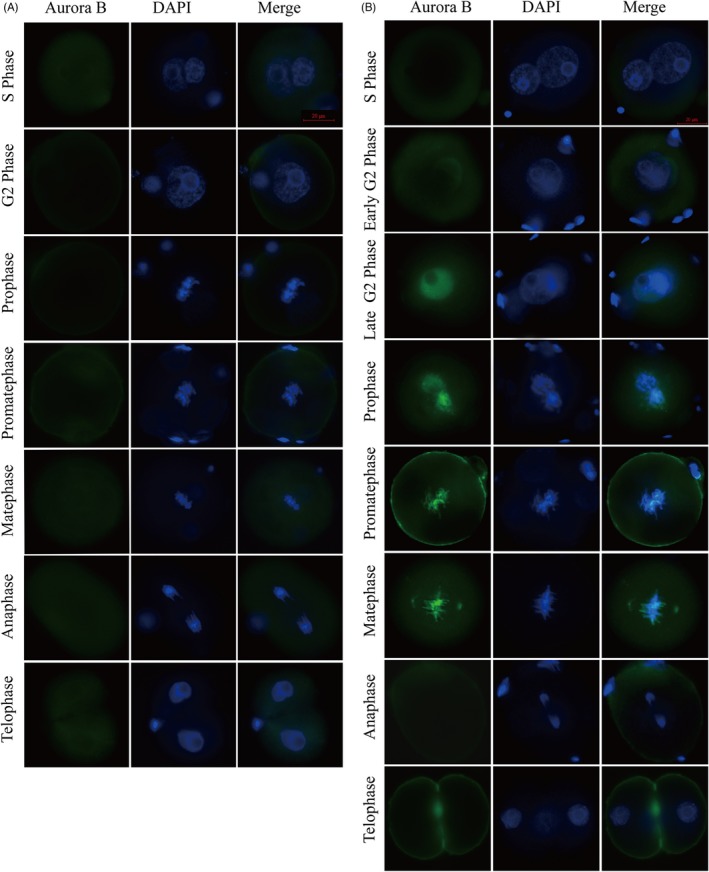
Immunofluorescence staining of Aurora B expression during various phases in IVF‐derived mouse embryos. A, There was no Aurora B (green) staining in the control group. B, In the H_2_O_2_‐treated control group, Aurora B (green) was not detected in the S phase in zygotes. Aurora B signal was observed in the nucleus in late G2 phase and in prometaphase and metaphase. During anaphase and telophase, the fluorescence signal of Aurora B rapidly disappeared. Nuclei were stained with DAPI (blue). The scale bar for the immunofluorescence images represents 20 μm

We first examined the subcellular localization of Aurora B during the first cleavage by immunostaining. Aurora B staining was not observed in the control group (Figure 1A). However, in H_2_O_2_‐treated group, we observed no Aurora B‐positive signals (green) during the S phase (18 hpi), whereas it was observed in the cytoplasm during early G_2_ phase and in the nucleus at late G_2_ phase (19 ~ 21 hpi), suggesting that oxidative DNA damage triggered nucleocytoplasmic transport of Aurora B. During prometaphase and metaphase (21.5 ~ 22.5 hpi), clear localization was detected in the chromatin, and the signal disappeared from the chromatin at anaphase and telophase (Figure 1B). These data imply that the SAC may contribute to cell cycle surveillance and that Aurora B is involved in the repair of oxidative stress‐induced DNA damage in mouse embryos during the first cleavage.

### 3.2 Inhibition of Aurora B by AZD1152‐HQPA caused arrest in IVF mouse embryos under mild oxidative damage

**Figure 2:**
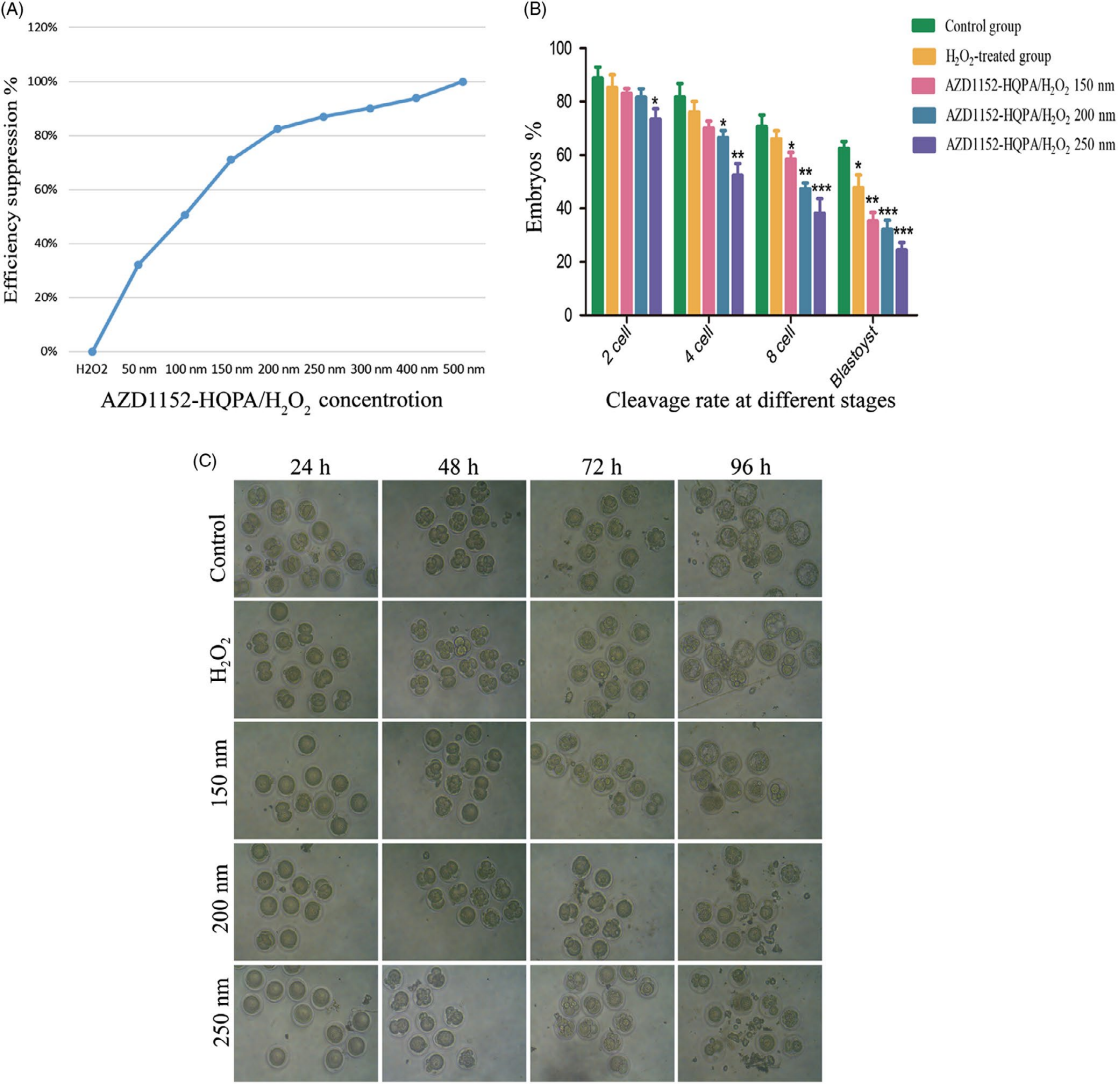
Comparison of embryo development between zygotes from groups treated with different concentrations of AZD1152‐HQPA. A, The Aurora B suppression efficiency of different concentrations of AZD1152‐HQPA/H_2_O_2_. *P < 0.05;**P < 0.01;***P < 0.001. B, Comparisons of cleavage rates from each group. There were observed a decrease in blastocyst cleavage rate in H_2_O_2_‐treated group (*P* < 0.05). However, addition of 200 nmol/L AZD1152‐HQPA significantly lowered the 4‐cell, 8‐cell and blastocyst formation rates, although no statistical differences were observed in the 2‐cell embryo formation rate (*P* > 0.05). Data are means ± standard deviations of three independent experiments. Differences between groups were calculated using chi‐square tests. (C) Representative images of embryos at 24, 48，72 and 96 hpi from each group, photographed under bright‐field conditions. Scale bar, 100 μm

To inhibit the function of Aurora B, zygotes were treated with different concentrations of AZD1152‐HQPA, a small molecule inhibitor of Aurora B^25^ and then oxidative DNA damage was induced with 0.03 mmol/L H_2_O_2_. The dose‐dependent effects of AZD1152‐HQPA on Aurora B positivity were then investigated (Table 1). Inhibition was found to be effective when the Aurora B positivity rate was less than 20%, and the Aurora B inhibition efficiency was more than 80%.^26^ The minimum effective concentration of AZD1152‐HQPA for the inhibition of Aurora B function in IVF‐derived embryos was 200 nmol/L (Figure 2A). Compared with the control group, H_2_O_2_ treatment did not significantly reduce the rates of formation of 2‐, 4‐ or 8‐cell embryos (*P* > 0.05), but did decrease the rate of blastocyst formation (*P* < 0.05). In contrast, inhibition of Aurora B reduced 4‐cell, 8‐cell and blastocyst formation rates compared with those from control and H_2_O_2_‐treated embryos (*P* < 0.05; Table 2; Figure 2B). As shown in Figure 2C, inhibition of Aurora B delayed cell division.

**Table 1:** Aurora B Suppression efficiency (%) and Aurora B‐positive rate in different concentrations of AZD1152‐HQPA under oxidative stress‐induced DNA damage in mouse embryos

Group	Aurora B‐positive zygotes/Total zygoted	Aurora B‐positive rate（%）	Aurora B suppression efficiency（%）
H_2_O_2_	47/125	37.6	0
50 nm	12/47	25.5	32.2
100 nm	8/43	18.6	50.5
150 nm	6/55	10.9	71.0
200 nm	4/61	6.6	82.4
250 nm	2/41	4.9	86.9
300 nm	3/81	3.7	90.1
400 nm	1/43	2.3	93.9
500 nm	0/40	0	100

**Table 2:** The development of zygotes in control group and 0.03 mmol/L H_2_O_2_‐treated group embryos and different concentrations of AZD1152‐HQPA group embryos at different stages

Group	2 cell (%)	4 cell (%)	8 cell (%)	Blastocyst (%)
Control	90.34 ± 3.79	81.39 ± 4.66	70.78 ± 2.92	62.64 ± 3.63
H_2_O_2_	85.24 ± 6.81	76.11 ± 5.50	65.95 ± 4.30	47.70 ± 6.87*
100 nm	83.66 ± 5.86	73.51 ± 7.28	59.29 ± 9.76	44.12 ± 9.82**
150 nm	82.96 ± 8.73	70.09 ± 3.59	54.47 ± 6.64*	35.23 ± 4.68***
200 nm	81.59 ± 4.59	63.91 ± 5.65*	41.82 ± 6.94**	32.17 ± 4.76***
250 nm	63.45 ± 5.44*	52.36 ± 6.35**	36.54 ± 7.03***	24.42 ± 3.97***
300 nm	50.44 ± 10.42**	31.36 ± 10.74***	14.76 ± 8.97***	0
400 nm	23.13 ± 9.12***	13.42 ± 7.50***	0	0

* *P* < 0.05

** *P* < 0.01

*** *P* < 0.001 compared with the control group.

### 3.3 Effects of Aurora B on oxidative stress‐related embryonic changes

#### 3.3.1 ROS concentrations in zygotes

**Figure 3:**
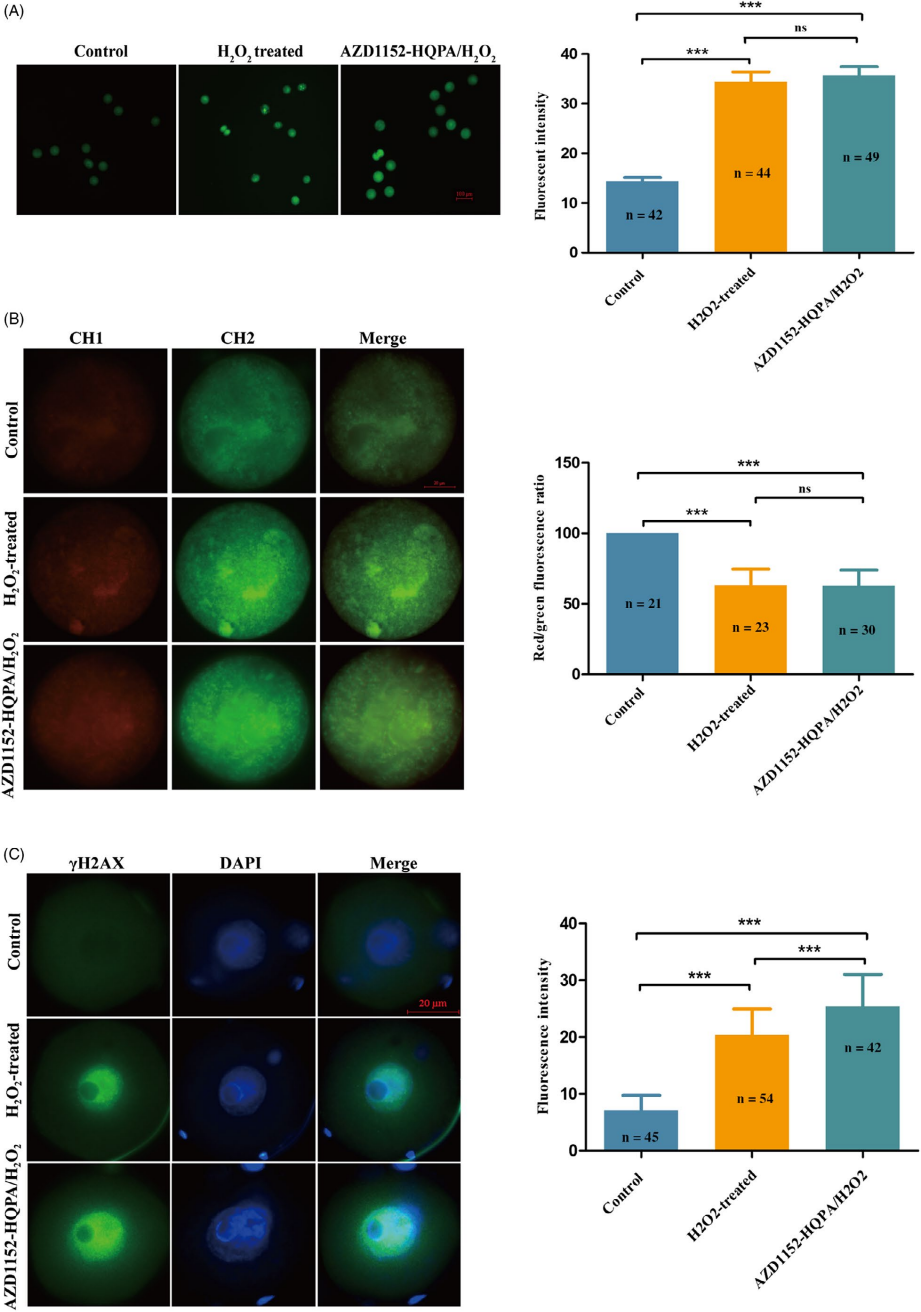
Comparison among groups of oxidative stress‐related embryonic changes. A, Representative images of ROS levels. Average fluorescence intensity per zygote. ***P < 0.001, H_2_O_2_‐treated group compared with the control group. ***P < 0.001, the 200 nmol/L AZD1152‐HQPA/H_2_O_2_‐treated group vs the control group, analysis of variance. B, Representative images of MMP in mouse zygotes are shown; red fluorescence from channel 1 represents J‐aggregates (highly polarized mitochondria); green fluorescence from channel 2 represents the monomer form of JC‐1 (weakly polarized mitochondria). Analysis of MMP via comparison of relative fluorescence intensities. The average value of red/green fluorescence intensity in the control group was set as 100%. ***P < 0.001, the H_2_O_2_‐treated group vs the control group. ***P < 0.001, the 200 nmol/L AZD1152‐HQPA/H_2_O_2_‐treated group vs the control group, analysis of variance. C, γH2AX detection in mouse zygotes. γH2AX (green) staining was detected in the nuclei of the H_2_O_2_‐treated and 200 nmol/L AZD1152‐HQPA/H_2_O_2_‐treated group, but not in those of the control group. Nuclei were stained with DAPI (blue). Scale bar, 20 μm. Analysis of the fluorescence intensity of γH2AX in each group. ***P < 0.001, H_2_O_2_‐treated group compared with the control group, analysis of variance. ***P < 0.001, the 200 nmol/L AZD1152‐HQPA/H_2_O_2_‐treated group compared with the H_2_O_2_‐treated group, Pearson chi‐square test. ns, no significance, *P < 0.05; **P < 0.01; ***P < 0.001

We next compared ROS generation in the control, H_2_O_2_‐treated and AZD1152‐HQPA/H_2_O_2_‐treated groups via 2′,7′‐dichlorofluorescein diacetate (DCFH‐DA) fluorescence analysis in zygotes (18 hpi) (Figure 3A). The mean fluorescence intensities of zygotes in the H_2_O_2_‐treated group (34.20 ± 12.22) and AZD1152‐HQPA/H_2_O_2_‐treated group (35.68 ± 11.86) were higher than that in the control group (14.31 ± 5.13; both *P* < 0.05). However, there were no significant differences in mean fluorescence intensity between the H_2_O_2_‐treated and AZD1152‐HQPA/H_2_O_2_‐treated groups (*P* > 0.05).

#### 3.3.2 Changes in mitochondrial membrane potential (MMP) in zygotes

To further characterize the adverse effects of mild oxidative stress on embryonic development in vitro, we monitored variations in MMP. The relative pixel‐intensity ratios of MMP in the H_2_O_2_‐treated group (63.15%±11.02%) and AZD1152‐HQPA/H_2_O_2_‐treated group (62.75%±10.87%) were lower than those in the control group (100%; both *P* < 0.001) (Figure 3B). However, there were no significant differences in MMP between the H_2_O_2_‐treated and AZD1152‐HQPA/H_2_O_2_‐treated groups (*P* > 0.05), indicating that Aurora B inhibition did not cause significant changes in MMP.

#### 3.3.3 γ‐H2AX‐fluorescence intensities in mouse zygotes

γH2AX is an early and sensitive marker of DNA damage. To confirm that H_2_O_2_ induces DNA damage, we monitored γH2AX‐fluorescence intensity by immunofluorescence in each group (Figure 3C). Compared with that in the control group, the γH2AX‐fluorescence intensity was increased in the H_2_O_2_‐treated group (7.12 ± 2.50 vs 20.41 ± 4.32; *P* < 0.001). Compared with the H_2_O_2_‐treated group, it was statistically higher in the AZD1152‐HQPA/H_2_O_2_‐treated group (20.41 ± 4.32 vs 25.94 ± 5.71; *P* < 0.001), suggesting that inhibition of Aurora B may aggravate embryonic DNA damage.

### 3.4 Inhibition of Aurora B by AZD1152‐HQPA caused chromosome misalignment and spindle destruction

**Figure 4:**
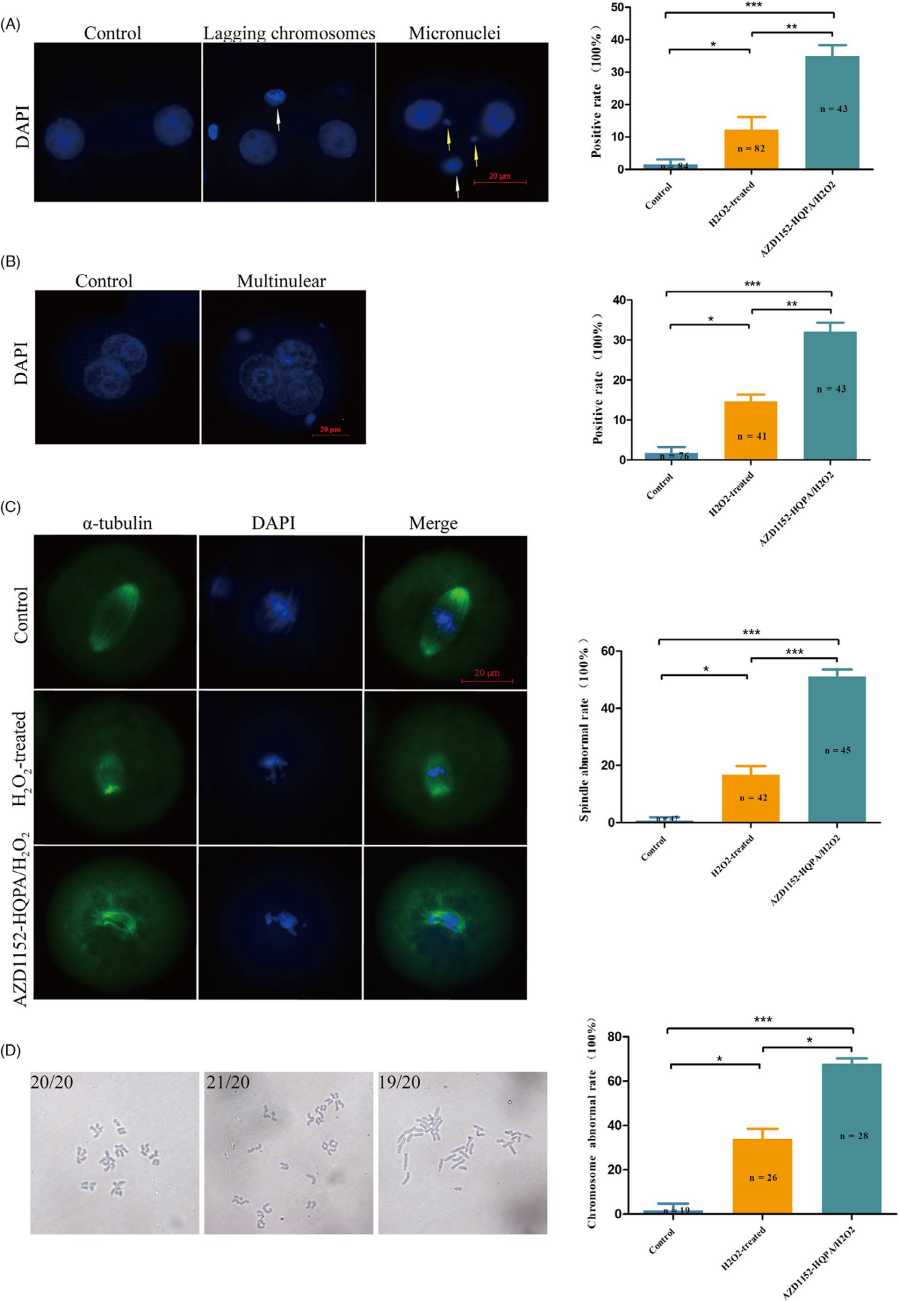
Inhibition of Aurora B by AZD1152‐HQPA caused abnormal chromosome segregation and spindle morphology. A, Representative images of micronuclei (white arrows) and lagging chromosomes (yellow arrows). Analysis of percentages of lagging chromosomes and/or micronuclei display in control, H_2_O_2_‐treated and 200 nmol/L AZD1152‐HQPA/H_2_O_2_‐treated groups. B, Multinuclear formation in treated zygotes indicated the occurrence of aneuploidy. Analysis of percentages of multinuclear in control, H_2_O_2_‐treated and 200 nmol/L AZD1152‐HQPA/H_2_O_2_‐treated groups. C, Localization of α‐tubulin was associated with spindle morphology during mitosis metaphase. Zygotes were stained with DAPI (blue) to detect nuclei and anti‐α‐tubulin antibodies (green) in metaphase. Control zygotes had a typical barrel‐shaped spindle at the metaphase. Malformed spindle in H_2_O_2_‐treated and 200 nmol/L AZD1152‐HQPA/H_2_O_2_‐treated groups. Scale bar, 20 μm. Analysis of percentages of abnormal spindle in control, H_2_O_2_‐treated and 200 nmol/L AZD1152‐HQPA/H_2_O_2_‐treated groups. D, Chromosome karyotype analysis in each group of IVF‐derived mouse embryos. Analysis of percentages of abnormal chromosome in control, H_2_O_2_‐treated and 200 nmol/L AZD1152‐HQPA/H_2_O_2_‐treated groups. (a) Normal (n = 20 bivalent); (b) hyperdiploidy (n = 21 bivalents); (c) hypodiploidy (n = 19 bivalents). *P < 0.05; ** *P* < 0.01; ***P < 0.001

We used DAPI staining to observe lagging chromosomes, micronuclei and multinuclei of IVF‐derived embryos through chromosome mis‐segregation during first mitosis. Our results showed that the rates of lagging chromosomes and/or micronuclear formation in the control group (2.4%, 2/84) was lower than those in the H_2_O_2_‐treated group (12.2%, 10/82; *P* < 0.05) and AZD1152‐HQPA/ H_2_O_2_‐treated group (34.9%, 15/43; *P* < 0.001). Compared with the H_2_O_2_‐treated group, it was statistically higher in the AZD1152‐HQPA/H_2_O_2_‐treated group (*P* < 0.01) (Figure 4A). Additionally, with regard to multinuclear formation, the rate in the control group (2.6%, 2/76) was lower than those in the H_2_O_2_‐treated group (14.6%, 6/41; *P* < 0.05) and AZD1152‐HQPA/H_2_O_2_‐treated group (39.5%, 17/43; *P* < 0.001). Compared with the H_2_O_2_‐treated group, it was statistically higher in the AZD1152‐HQPA/ H_2_O_2_‐treated group (*P* < 0.01) (Figure 4B).

We next examined the localization of α‐tubulin by immunostaining to determine abnormal spindle formation rates (Figure 4C). Normal spindle formation results in a bulge in the middle of the cell, with a spindle shape formed at both ends.^27^ Our results showed that the abnormal spindle formation rate in the control group (2.1%, 1/47) was lower than those in the H_2_O_2_‐treated group (16.7%, 7/42; *P* < 0.05) and AZD1152‐HQPA/ H_2_O_2_‐treated group (51.1%, 23/45; *P* < 0.001). Compared with the H_2_O_2_‐treated group, it was statistically higher in the AZD1152‐HQPA/ H_2_O_2_‐treated group (*P* < 0.001).

Next, we examined the chromosome karyotype of IVF embryos to analyse the number of chromosomes during mitosis (Figure 4D). Our results showed that the abnormal chromosome number rate in the control group (5.2%, 1/19) was lower than those in the H_2_O_2_‐treated group (34.6%, 9/26; *P* < 0.05) and AZD1152‐HQPA/ H_2_O_2_‐treated group (67.8%, 19/28; *P* < 0.001). Compared with the H_2_O_2_‐treated group, it was statistically higher in the AZD1152‐HQPA/ H_2_O_2_‐treated group (*P* < 0.05).

### 3.5 Aurora B participates in SAC regulation of chromosome segregation during the first mitotic division in oxidative DNA damage in IVF‐derived embryos

#### 3.5.1 Colocalization of Aurora B and Mad2 during various phases in IVF‐derived zygotes

**Figure 5:**
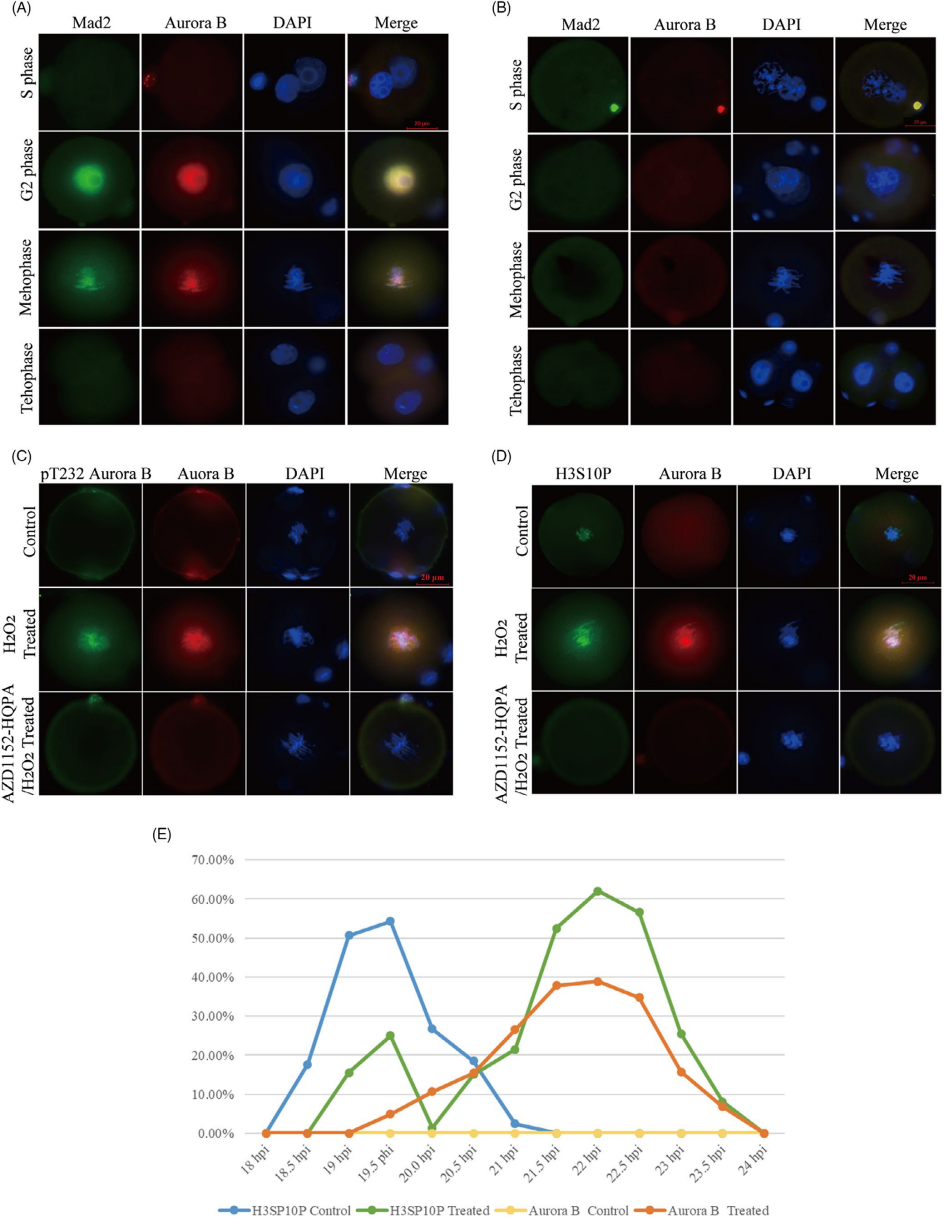
Immunofluorescence staining in IVF‐derived mouse embryos. A, Colocalization of Aurora B (red) and Mad2 (green) was detected in H_2_O_2_‐treated group. The localization of Mad2 exhibited similar patterns as Aurora B expression. B, Neither Aurora B (green) nor Mad2 (red) was detected in the 200 nmol/L AZD1152‐HQPA/H_2_O_2_‐treated group. C, Colocalization phospho‐Thr232 Aurora B (green) and Aurora B(red) was detected in H_2_O_2_‐treated group, phospho‐Thr232 Aurora B (green) and Aurora B(red) were not detected in the control group or 200 nmol/L AZD1152‐HQPA/H_2_O_2_‐treated group. D, Colocalization of H3S10P (green) and Aurora B (red) in IVF‐derived mouse embryos. H3S10P (green) was detected but Aurora B (red) was not detected in the control group. H3S10P (green) and Aurora B (red) was detected in H_2_O_2_‐treated group. Neither H3S10P (green) nor Aurora B (red) was detected in the 200 nmol/L AZD1152‐HQPA/H_2_O_2_‐treated group. Nuclei were stained with DAPI (blue). Scale bar, 20 μm. E, Percentage of cells showing H3S10P expression in control and H_2_O_2_‐treated zygotes from 18 to 24 hpi

Aurora B is required for kinetochore localization of the spindle checkpoint component Mad2 in prometaphase and metaphase.^26^, ^27^, ^28^ We analysed the subcellular colocalization of Mad2 and Aurora B in response to oxidative DNA damage in IVF‐derived embryos. In H_2_O_2_‐treated zygotes, Aurora B signals were observed in the nucleus at late G_2_ phase. During prometaphase and metaphase, Aurora B was enriched in the chromatin, whereas during anaphase and telophase, when the correct kinetochore‐microtubule attachment had been established, the Aurora B signal disappeared. Mad2 was enriched in the chromatin at late G_2_ phase and metaphase, similar to the localization of Aurora B (Figure 5A). However, in the Aurora B‐inhibited zygote and control groups, there were no Aurora B and Mad2 signals in the chromatin (Figure 5B).

We further analysed the possible relationship between Aurora B and the SAC key protein Mad2 in IVF‐derived zygotes. Our results showed that the rates of Aurora B‐Mad2 foci were enriched in the chromatin in the H_2_O_2_‐treated group (38.9%, 23/59) was higher than those in the control (2.2%, 1/46) and AZD1152‐HQPA/H_2_O_2_‐treated groups (6.6%, 4/61, both *P* < 0.001). Furthermore, inhibition of Aurora B resulted in an 83.0% reduction of Aurora B‐Mad2 localization in the AZD1152‐HQPA/H_2_O_2_‐treated group. However, there was no significant difference between the control group and the AZD 1152‐HQPA/ H_2_O_2_ treatment group (*P* > 0.05), which are indicative of Aurora B may affects the localization of Mad2.

#### 3.5.2 Colocalization of phospho‐Thr232 Aurora B and Aurora B demonstrated enhanced Aurora B activation

Phosphorylation of Thr232 is necessary for Aurora B activation. Therefore, we examined the expression and subcellular colocalization of phospho‐Thr232 Aurora B and Aurora B(red) during the first mitosis by immunostaining. While no phospho‐Thr232 Aurora B (green) or Aurora B(red) staining was observed in the control and AZD1152‐HQPA/H_2_O_2_‐treated group. In H_2_O_2_‐treated group, the fluorescent signals of phospho‐Thr232 Aurora B (green) and Aurora B(red) were detected in the chromatin at metaphase (Figure 5C).

#### 3.5.3 Colocalization of H3S10P and Aurora B in IVF‐derived zygotes

Our previous experiments showed that H3S10P in whole chromatin is indicative of prometaphase/metaphase delay and SAC activation under oxidative stress during the first mitotic division.^29^ Thus, we performed immunofluorescence staining to determine the colocalization of H3S10P (green) and Aurora B(red) in each group. In control group, H3S10P (green) was observed in the chromatin at metaphase, whereas Aurora B (red) was not detected. In H_2_O_2_‐treated zygotes, the fluorescence signal of H3S10P (green) was observed in the chromatin at metaphase, and the localization of Aurora B (red) was similar to that of H3S10P. In contrast, H3S10P and Aurora B were not expressed in the AZD1152‐HQPA/H_2_O_2_‐treated group (Figure 5D). We monitored the ratios of positive zygotes by immunofluorescence staining for H3S10P and Aurora B at 30‐min intervals from 18 to 24 hpi (Table 3). The results showed that the percentage of H3S10P‐positive zygotes in the control group was above 50% at 19.0 and 19.5 hpi (50.70% and 54.3%, respectively), reaching a maximum at 19.5 hpi and decreasing to zero at 21.0 hpi (ie, when zygotes finished the first cleavage). There was no Aurora B signal in the control group. However, in the H_2_O_2_‐treated group, the percentage of H3S10P‐positive zygotes was above 50% at 21.5, 22.0 and 22.5 hpi (52.5%, 62.1% and 56.6%, respectively). Moreover, the percentage of Aurora B‐positive zygotes was similar to that of H3S10P‐positive zygotes, reaching a maximum at about 22 hpi (Figure 5E). These findings indicate that Aurora B induces a prometaphase/metaphase delay under oxidative stress during the first mitotic division in IVF‐derived zygotes.

**Table 3:** Ratio of H3S10P and Aurora B–expressing cells in control and treated zygotes from 18 hpi to 24 hpi

Groups	18 hpi	18.5 hpi	19.hpi	19.5 phi	20.0 hpi	20.5 hpi	21 hpi	21.5 hpi	22 hpi	22.5 hpi	23 hpi	23.5 hpi	24 hpi
H3S10P Control	0/43	9/51	34/67	38/70	19/71	12/65	1/41	0	0	0	0	0	0
H3S10P Treated	0/49	0/42	7/45	19/76	1/71	9/60	11/51	32/61	36/58	30/53	13/51	5/62	0
Aurora B Control	0	0	0	0	0	0	0	0	0	0	0	0	0
AuroraB Treated	0/40	0/46	0/51	2/41	5/47	8/52	21/79	28/74	37/95	31/89	11/70	4/59	0

Note

The treated group was administered 0.03 mmol/L H_2_O_2_.

Abbreviation: Hpi, hours post‐insemination.

### 3.6 Oxidative stress–induced Aurora B signalling pathway

**Figure 6:**
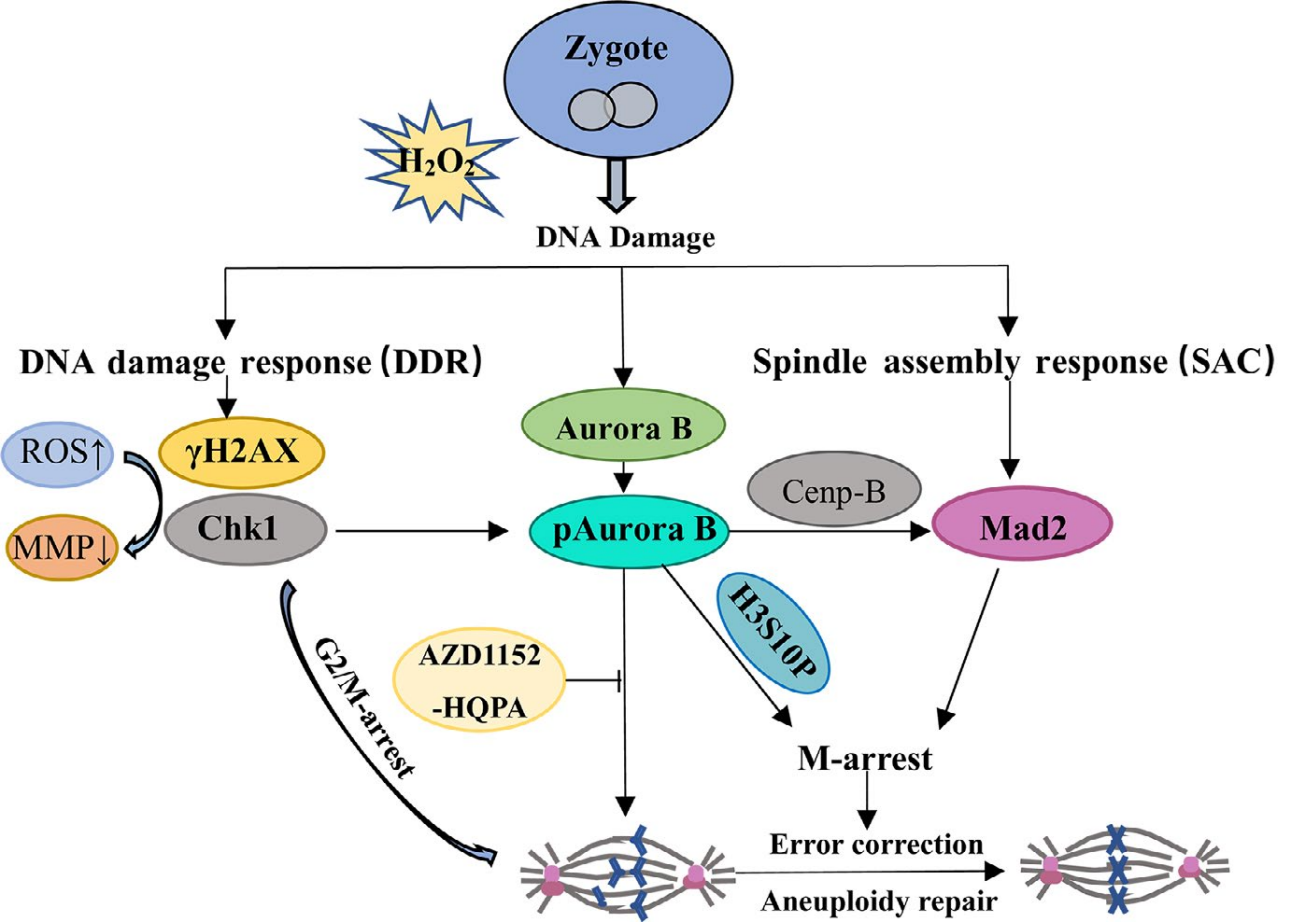
Oxidative stress‐induced Aurora B signalling pathway. Schematic representation shows of a model for Aurora B promotion of aneuploidy repair via Mad2‐mediated activation of SAC

A schematic representation of the mechanism by which Aurora B prevents aneuploidy via Mad2‐mediated activation of SAC is given in Figure 6. DNA damage induced by oxidative stress triggers two surveillance mechanisms: the DNA damage response (DDR) and SAC. DDR and SAC evoke cell cycle arrest in G2/M and M phases and are responsible for DNA repair and chromosomal stability, respectively. Chk1 is presented to its upstream regulatory protein, Aurora B, which is then activated in response to DNA damage.^21^ Inhibition of Aurora B by AZD1152‐HQPA leads to chromosomal misalignment, which in turn cause activation of Aurora B in response to error correction.

## 4 DISCUSSION

Chromosome aneuploidy, which leads to pre‐implantation embryo arrest, is the main reason for the failure of IVF embryo implantation.^18^ To understand the high failure rate of aneuploidy, we investigated the associated mechanisms of aneuploidy in IVF‐derived embryos. DDR and SAC are together responsible for DNA repair and chromosomal stability after oxidative stress‐induced DNA damage.^30^, ^31^, ^32^

Aurora B plays a role in the spindle checkpoint dependent of its error correction function in many mitotic processes.^33^, ^34^ In mouse early embryos in vitro, to determine whether Aurora B plays a role during cleavage divisions in zygotes, we first observed the localization of Aurora B in H_2_O_2_‐treated zygotes. Our previous studies revealed that the zygote cell cycle checkpoint was activated by oxidative stress–induced DNA damage and delayed cell cycle entry, causing M‐phase delay.^29^ The mitotic cell cycle checkpoint is delayed until all kinetochores are properly attached to the spindle to protect against and prevent erroneous chromosome segregation.^35^ Thus, our observations indicated that Aurora B plays a key role in regulating SAC during the first cleavage of IVF‐derived embryos under oxidative stress.

Because of this, Aurora B is a key regulator for cytoplasmic division and proper chromosome segregation,^36^ and the loss of Aurora B is an important cause of SAC slippage and chromosomal abnormalities during mitosis.^37^ In this study, we observed that AZD1152‐HQPA/H_2_O_2_‐treated zygotes exhibited delayed cell division, impairing cell cycle progression. Consistent with this, we found that inhibition of Aurora B affected early embryonic development during the first mitotic cleavage in mouse embryos.

Oxidative DNA damage is one of the main reasons for the arrest and death of early embryos.^38^ Our results showed higher levels of ROS and decreased MMP in zygotes affected by oxidative stress during in vitro culture. However, Aurora B inhibition did not cause significant changes in ROS or MMP, demonstrating that zygotes are affected by oxidative stress during in vitro culture. Moreover, Aurora B inhibition caused a significant increase in γH2AX‐positive cells compared with that in the H_2_O_2_‐treated group, indicating that inhibition of Aurora B may aggravate embryonic DNA damage.

In mitotic cells, Aurora B promotes proper chromosome segregation in part by regulating chromosome alignment at the metaphase plate; this may function to ensure embryonic euploidy.^39^, ^40^ Disruption of Aurora B function leads to chromosome segregation defects, including nondisjunction, lagging chromosomes and cytokinesis failure.^41^ The frequent observation of micronuclei in embryos suggests defects in chromosome stability.^42^ In this study, we found that Aurora B inhibition in IVF embryos causes a significant increase in abnormal chromosome segregation, such as lagging chromosomes and micronuclear/multinuclear formation. Accordingly, our results demonstrate that Aurora B plays key role in regulating chromosome alignment and in the processes of attachment‐error correction during the first mitotic division in IVF embryos under oxidative stress.

During mitosis, the formation of the spindle structure is essential for chromosome segregation and cytokinesis.^43^ Utilizing immunofluorescence, we showed that two bipolar spindles without clear end boundaries formed in Aurora B‐inhibited zygotes and that the midbody did not have an normal structure. Our current results showed that AZD1152‐HQPA/H_2_O_2_ treatment also led to a high incidence of spindle structure defects. Such defects have the potential to generate aneuploidy, suggesting that Aurora B has a functional role in mediating correct bipolar spindle formation in IVF‐derived embryos during mitosis.

Aneuploidy is the most common chromosomal abnormality, and chromosome segregation errors can lead to chromosome structure and number alterations,^44^, ^45^ eventually causing embryo arrest or severe developmental defects.^46^ Therefore, we here used chromosome karyotype analysis to detect aneuploidy during cleavage in the embryos in each group. This analysis revealed that the incidence of abnormal chromosome number was increased in the AZD1152‐HQPA/H_2_O_2_‐treated group. Our results show that the chromosome instability caused by Aurora B inhibition in IVF embryos may lead to an increase in the incidence of aneuploidy. This suggests that Aurora B may be involved in the self‐correction of chromosome aneuploidy during the first mitosis in IVF‐derived embryos.

Aurora B has been well studied in relation to SAC and is required for the recruitment of SAC components, such as Mad2, to the kinetochore in prometaphase.^26^, ^47^ Our previous experiments demonstrated that Mad2 localizes to the kinetochore in zygotes under oxidative stress.^21^ Additionally, Aurora B localizes to kinetochores and regulates kinetochore‐microtubule interactions in the first mitotic division.^48^, ^49^ In this study, we aimed to determine whether Aurora B is required for the recruitment of the SAC key protein Mad2 to unattached kinetochores in IVF‐derived mouse early embryos. Our results showed that Aurora B‐Mad2 colocalized in the chromatin in response to oxidative DNA damage, the inhibition of Aurora B resulted in no detectable Mad2 in the chromatin in zygotes, suggesting that Aurora B inhibition affects the localization of Mad2, both of which cause SAC defects and a high incidence of aneuploidy under oxidative DNA damage. In this study, we showed that Aurora B is required for Mad2 localization to kinetochores, where it participates in SAC to regulate chromosome segregation and prevent aneuploidy events associated with the first cleavage in zygotes under oxidative stress.

The phosphorylation of Aurora B at Thr232 is an essential regulatory mechanism for Aurora B activation.^50^ Here, our results showed that the phosphorylation of Aurora B at Thr232 increased kinase activity, which provided further evidence that Aurora B is involved in SAC activation following oxidative damage in zygotes. Aurora B is responsible for the phosphorylation of histone H3 at Ser10 (H3S10P) during mitotic division,^51^ and H3S10P has been shown to be a marker of Aurora B activity.^52^, ^53^ H3S10P is involved in the first mitotic division and affects cell cycle progression during mitotic division in porcine embryos.^53^ Our results showed that the role of Aurora B was likely tied to its interaction with H3S10P in the chromatin delaying the first mitotic division to self‐correct chromosome abnormalities in response to oxidative DNA damage in IVF‐derived embryos. Furthermore, the percentage of Aurora B‐positive zygotes was similar to that of H3S10P‐positive zygotes peaking at metaphase. This further indicated that Aurora B regulates the first mitotic division via histone H3 phosphorylation, providing more time for the self‐correction of chromosome misarrangements during the first mitotic division in mouse pre‐implantation embryos. Thus, Aurora B plays an important role in the prevention of chromosome aneuploidy and the reduction of the implantation failure rate of IVF embryos.

In conclusion, aneuploidy formation can arise from errors in chromosome segregation or spindle distribution, causing embryo implantation failure. Aurora B affects the mitotic checkpoint dependent of its role in destabilizing incorrect kinetochore‐microtubule attachments. We found that Aurora B in in vitro culture affects the expression of Mad2, a key SAC protein, spindle morphology and chromosome alignment. Furthermore, our results indicate that it potentially impairs the ability to repair DNA damage. Our findings support the idea that Aurora B is a key regulator of mitosis for the self‐correction of chromosome abnormalities during embryonic development and may facilitate the development of assisted reproductive technologies.

## CONFLICT OF INTERESTS

The authors declare that they have no conflict of interests.

## AUTHOR CONTRIBUTIONS

ZLL had the initial idea, supervised experiments and data analysis, and revised the manuscript. JNL and SYH designed the experiments and the statistical analysis. JNL, SYH, ZLL, YH, EL and WFX performed the experiments and wrote the manuscript. All authors have read the manuscript and approved the final version.

## Data Availability

The data used to support the findings of this study are available from the corresponding author upon request.
